# The emerging role of the KCTD proteins in cancer

**DOI:** 10.1186/s12964-021-00737-8

**Published:** 2021-05-17

**Authors:** Annapaola Angrisani, Annamaria Di Fiore, Enrico De Smaele, Marta Moretti

**Affiliations:** 1 Department of Molecular Medicine, Sapienza University of Rome, Rome, Italy; 2 Department of Experimental Medicine, Sapienza University of Rome, Rome, Italy

**Keywords:** KCTD family, BTB domain, Cancer, Oncogene, Tumor suppressor, KCASH family, KCTD15, Cul3, Ubiquitination

## Abstract

The human family of *Potassium (K*+*) Channel Tetramerization Domain* (KCTD) proteins counts 25 members, and a significant number of them are still only partially characterized. While some of the KCTDs have been linked to neurological disorders or obesity, a growing tally of KCTDs are being associated with cancer hallmarks or involved in the modulation of specific oncogenic pathways. Indeed, the potential relevance of the variegate KCTD family in cancer warrants an updated picture of the current knowledge and highlights the need for further research on KCTD members as either putative therapeutic targets, or diagnostic/prognostic markers. Homology between family members, capability to participate in ubiquitination and degradation of different protein targets, ability to heterodimerize between members, role played in the main signalling pathways involved in development and cancer, are all factors that need to be considered in the search for new key players in tumorigenesis. In this review we summarize the recent published evidence on KCTD members’ involvement in cancer. Furthermore, by integrating this information with data extrapolated from public databases that suggest new potential associations with cancers, we hypothesize that the number of KCTD family members involved in tumorigenesis (either as positive or negative modulator) may be bigger than so far demonstrated.

**Video abstract**

**Supplementary Information:**

The online version contains supplementary material available at 10.1186/s12964-021-00737-8.

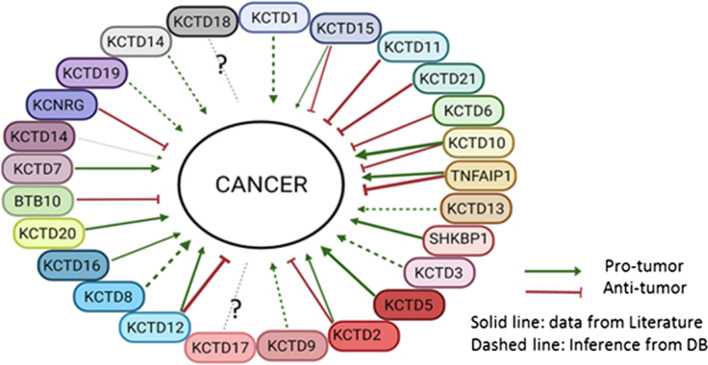

## Background

The human family of *Potassium (K*+*) Channel Tetramerization Domain* (KCTD) proteins counts 25 members which are only partially characterized, although their increasing relevance in various important biological functions is being gradually uncovered [1]. Indeed, ever-growing evidences are pointing to a significant role of this diverse family in mechanisms of protein degradation and other biological functions, hinting for several of them as potential players in cancer development or cancer prevention, as well as potential therapeutic targets in the treatment of tumor.

The KCTD family arose from a common ancestral gene by gene duplication and evolutive divergence and share a conserved domain, called BTB (Broad complex, Tramtrak and Bric‐a‐brac)/POZ (poxvirus zinc finger) domain [2, 3]. This domain (from now on BTB domain) is a simple protein motif (approximately 95 amino acids) which is functional for protein oligomerization [4] and to establish protein–protein interactions and is essential for the diverse biological functions of the KCTD family members [5].

Comparative analyses of the full aminoacidic sequences of all family members suggested that KCTD proteins can be clustered in seven clades, each composed of KCTD paralogs that often share similar biological functions [6]. However, a more recent classification from Teng et al., based on the minimal BTB domain sequences alignment and the N-terminal domain structures, subdivides the KCTD proteins into eight groups [1]. In this review, we will use this latter classification. We also refer to Teng et al.’s work for the available details on binding partners, BTB structures and additional protein domains of the KCTD family.

In a first attempt of characterization, KCTD family members can be classified according to the capability of binding to E3-ubiquitin ligases, through their BTB domain, and thus participate in degradative processes. Indeed, KCTD proteins of group B (KCASH1^KCTD11^, KCASH2^KCTD21^, KCASH3^KCTD6^), group C (KCTD10, -13, TNFAIP1), group D (KCTD3, SHKBP1), group E (KCTD2, -5, -9, -17) and group H (KCTD7, -14) can form a E3-ubiquitin ligase complex together with Cullin RING E3 Ligases (CRL, most frequently partnering with Cullin3 (Cul3) ligase).

Most KCTD proteins act therefore as adapters, recruiting selectively substrates for ubiquitination [4, 7–13].

On the other hand, CRL recruitment is not a property present in all KCTD proteins [14]: indeed, some KCTD, belonging to group A (KCTD1 and KCTD15), group F (KCTD8, -12, -16) and group G (KCTD20, BTBD10) seem to have Cullin-independent functions [6, 9, 15, 16].

So far, KCTD genes have been associated with several diseases, including neurodevelopmental, neuropsychiatric, and neurodegenerative disorders [1, 17]. More recently, several KCTD have also been associated to cancer (see below) although the identification of KCTD-containing players in cancer appears far from completed.

In this review, we aim to discuss the role of KCTD proteins in cancer, analyzing the published literature and adding further insights, by collecting evidences based on differential gene expression and presence of genic mutations in various tumor tissues obtained from two different databases (db), COSMIC (Catalogue Of Somatic Mutations In Cancer; https://cancer.sanger.ac.uk/cosmic [18, 19] and GENT2 (Gene Expression patterns across Normal and Tumor tissues; http://gent2.appex.kr/gent2/ [20].

COSMIC is the largest well annotated collection of somatic variants detected in tumor samples. A considerable fraction of COSMIC data is generated by whole genome screens, making them suitable for comparative analysis of mutability of human genes.

Gene expression data were analyzed through the Gene Expression database of Normal and Tumor tissues (GENT2) and based on the GEO public repository using the U133Plus2 (GPL570) platform and we focused on the information that was statistically significant.

Both db classify tumors according to their anatomic localization. While differential expression of a gene in a specific tumor type is generally consistent in published data (when available) and in db data, sometimes differences may occur; discrepancies are probably due to the existence of histological and molecular subgroups in the different tumor types, which are often not considered in these large databases. For this reason, we have taken in account in first approximation only data which are statistically significant (GENT) and consistent between the two db.

In the next sections we will first present the KCTD groups that have greater evidence of implication in tumorigenesis. Then, we will explore groups which, although do not appear yet to have strong experimental evidence, may still have a role in cancer, either because they are involved in signaling pathway known to lead to tumorigenesis or because of new hints obtained from our current databases observations.

An overview of the evidence on KCTD family’s role in cancer and the pathways involved is summarized in Table 1.

**Table 1:** Differential expression of KCTD family members in human tumors

Group	Gene	Cancer	Literature data		Cosmic		GENT2	
			Expression	Molecular target	Gene expression	Frequency	FC (T/N tissue)	*P* value
Group C	KCTD10	Burkitt's lymphoma	O/E [29]					
		Lymphoblastic leukemia	O/E [29]					
		Colorectal adenocarcinoma	O/E [29]					
		Gastrointestinal stromal tumor	O/E [37]					
		Lung carcinoma	O/E [29]					
		Ovarian cancer	O/E [27]					
		Melanoma	O/E [29]					
		Breast cancer	O/E [25]	RhoB, Rac1				
		Pancreatic cancer	O/E [28]					
		Hepatocellular carcinoma	U/E [38]	EIF3D				
		Adrenal gland cancer			O/E	5/79 (6.3%)	1.41	< 0.001
	TNFAIP1	Osteosarcoma	O/E [43, 52]	NF- κB signaling				
		Breast cancer	O/E [53]					
		Hepatocellular carcinoma	U/E [41]	CSNK2B				
		Non-small cell lung cancer	U/E [46, 47]					
		Uterus cancer	U/E [49]					
		Pancreatic cancer	U/E [48]					
		Gastric carcinoma	U/E [42–45]					
		Cervical carcinoma	U/E [49, 50]	RhoB				
		Adrenal gland cancer			U/E	11/79 (13.9%)	0.85	< 0.05
	KCTD13	Breast cancer			O/E	154/1104 (13.9%)	1.85	< 0.001
Group B	KCTD11	Medulloblastoma	U/E [59, 61, 62]	Hedgehog signaling				
		Endometrium cancer	U/E [61]					
		Gallbladder cancer	U/E [61]					
		Urinary bladder cancer	U/E [61]					
		Colorectal cancer	U/E [61]					
		Stomach cancer	U/E [61]					
		Lung cancer	U/E [61]					
		Larynx cancer	U/E [61]					
		Breast cancer	U/E [61]					
		Esophageal cancer	U/E [61]					
		Prostate carcinoma	U/E [59, 61, 62]					
		Hepatocellular carcinoma	U/E [63]	MST1/GSK3β/p21 signaling				
		Ovarian cancer			U/E	159/266 (59.8%)	0.82	
	KCTD21	Medulloblastoma	U/E [12, 15, 66]	Hedgehog signaling				
	KCTD6	Medulloblastoma	U/E [12, 15, 66]	Hedgehog signaling				
Group A	KCTD15	Lymphoblastic leukemia	O/E [80]					
		Myeloid leukemia	O/E [80, 81]					
		Medulloblastoma	U/E [15]	Hedgehog signaling				
	KCTD1	Ovarian cancer			O/E	37/266 (13.9%)	1.47	< 0.001
		Endometrium cancer			O/E	59/600 (9.8%)	1.43	< 0.001
		Pancreatic cancer			O/E	20/179 (11.17%)	1.32	< 0.001
		Lung cancer			O/E	76/1019 (7.46%)	1.21	< 0.001
Group D	SHKBP1	Small intestine neuroendocrine tumor	O/E [88]					
		Osteosarcoma	O/E [86]	EGFR signaling				
		Myeloid leukemia	Mut.V89I [89]	FLT3 Tyrosine kinase				
		Cervical cancer	Mut [90]		O/E	33/307 (10.8%)	2.14	< 0.05
		Pancreatic cancer			O/E	24/179 (13.4%)	2.05	< 0.001
		Nervous system tumor			Mutation	7/130 (5.4%)		
		Large intestine tumor			Mutation	113/2513 (4.5%)		
	KCTD3	Stomach cancer			O/E	37/285 (13.1%)	1.83	< 0.001
		Breast cancer			O/E	157/1104 (14.2%)	1.3	< 0.001
		Nervous system tumor			Mutation	7/130 (3.9%)		
Group F	KCTD12	Cervical cancer	O/E [102, 103]	CDC25B, CDK1				
		Lung cancer	O/E [102, 103]	CDC25B, CDK1				
		Gastrointestinal stromal tumor	U/E [96, 97]					
		Colorectal cancer	U/E [98, 102, 103]	CDK1				
		Uveal melanoma	U/E [100]					
		Esophageal squamous cell carcinoma	U/E [101]	WNT, Notch signaling				
		Breast cancer	U/E [99]	FoxO/Akt signaling				
	KCTD8	Cervical cancer			O/E	17/307 (5.5%)	n.a	
		Pancreatic cancer			O/E	13/179 (7.3%)	n.a	
		Esophageal cancer			O/E	9/125 (7.2%)	n.a	
		Thyroid cancer			O/E	41/513 (8.0%)	n.a	
	KCTD16	Small cell lung cancer	O/E [104]					
		Thyroid cancer	O/E [105]					
		Hepatic cancer			Mutation	296/2216 (13.6%)		
		Pancreatic cancer			Mutation	216/1840 (11.7%)		
		Prostate carcinoma			Mutation	177/1984 (8.9%)		
Group E	KCTD5	Melanonoma	O/E [110]	Rac1, Ca2 + signaling				
		Breast cancer	O/E [110, 111]	TRPM4, Rac1, Ca2 + signaling	O/E	140/1104 (12.5%)	1.33	< 0.001
	KCTD2	Colonrectal cancer	O/E [115]					
		Glioma	U/E [8]	C-Myc				
	KCTD9	Stomach cancer			O/E	42/285 (14.7%)	1.25	< 0.05
Group G	KCTD20	Non-small cell lung cancer	O/E [113]	Akt signaling, E-cadherin				
	BTBD10	Glioma	U/E [120, 122]	Akt signaling				
Group H	KCTD7	Glioblastoma	O/E [123]					
		Lung adenocarcinoma	O/E [124]					
		Adrenal gland cancer			O/E	11/79 (13.9%)	1.61	< 0.001
		Skin cancer			O/E	40/473 (8.46%)	2.28	< 0.001
		Breast cancer			O/E	68/1104 (6.16%)	1.16	< 0.001
		Pancreatic cancer			Mutation	9/165 (5.5%)		
	KCTD14	Ovarian cancer			CNV gain	31/684 (4.5%)	1.53	< 0.001
N.C	KCNRG	Chronic lymphocitic leukemia	U/E [126]					
		Multiple myelomas	U/E [126]					
		Hepatocellular carcinoma	Mut [127]					
	KCTD19	Hematopoietic and lymphoid tumor			O/E	28/221 (12.7%)	1.14	< 0.001
		Nervous system tumor			Mutation	6/130 (4.7%)		
		Large intestine tumor			Mutation	89/2502 (3.2%)		
		Skin cancer			Mutation	76/1279 (4.2%)		
	KCTD4	Lung cancer			O/E	51/1019 (5.0%)	1.32	< 0.001

Summary of the KCTD family members and the evidence of their involvement (or differential expression) in tumors, as reported in literature. Where possible, data suggesting further roles for KCTD members in tumors, extrapolated from COSMIC db (in terms of gene expression and frequency of tumor samples with overexpression, underexpression or mutation of the gene of interest compared to total analysed tumor samples) and GENT2 db (in numerical terms of FC = fold change, calculated from the ratio of expression in tumor and normal tissues, and statistical ones of p-value) are also indicated. Numbers in brackets in the “Expression” column indicate the relative bibliographic references; O/E: overexpression; U/E: underexpression; n.a.: not available.

## The KCTD proteins of group C: oncogenes or tumor suppressors?

KCTD10, TNFAIP1 (Tumor Necrosis Factor Induced Protein 1, also known as B12 or Bacurd2) and KCTD13 (also known as Bacurd1) proteins, belong to Group C. In addition to the capability of binding Cul3 ubiquitin ligase and thus inducing degradation of some interacting partners, this group is characterized by a proliferating cell nuclear antigen (PCNA)-binding motif at the C-terminus [1, 21]. Indeed, they are TNF-α/IL-6 -inducible proteins which are able to stimulate the activity of DNA polymerase δ in presence of PCNA, playing a role in DNA replication, repair and cell cycle control [22]. Given the pleiotropic role of TNF in immune response and inflammation [23] and the panoply of biological processes in which these KCTD proteins may be involved, their differential expression between normal and cancer tissues is not surprising.

***KCTD10*** gene maps to chromosome 12q24.11 and is strongly conserved from zebrafish to human [24]. Its expression is detectable in all human tissues with higher levels in heart, skeletal muscle, and placenta. In HER2‐positive breast cancers, one of the most aggressive subtypes of breast cancer, KCTD10 induces RhoB degradation and activation of Rac1 [25], which promotes progression of tumors, and resistance to therapy [26]. Similarly, KCTD10 expression has been associated to unfavorable prognosis in patients with early-stage clear-cell ovarian carcinoma [27] suggesting that in these contexts KCTD10 may be a therapeutic target.

Moreover, KCTD10 expression results upregulated, and it has been indicated as key to the pancreatic carcinogenesis [28].

High KCTD10 expression levels have also been detected in lymphoblastic leukemia, Burkitt’s lymphoma, colorectal adenocarcinoma, lung carcinoma and melanoma tumor lines [29]; nevertheless, the KCTD10 involvement in these tumors has yet to be investigated in detail.

Analysis of the COSMIC and GENT db confirm observations in melanoma, but also suggests KCTD10 overexpression in adrenal gland tumors, in a 6.3% of cancer tissues analyzed. Similarly, GENT2 db indicates a gene expression ratio of KCTD10 in adrenal tumor vs normal tissues [FC (fold change) = 1.41 (*p* value < 0.001)], suggesting the involvement of KCTD10 in this tumor type.

Ren and colleagues, through the generation of a KCTD10^KO^ mouse model, have demonstrated that KCTD10 could be involved in embryonic angiogenesis and heart development by negatively regulating the Notch signaling pathway, via Notch1 proteolytic degradation [30]. The role of KCTD10 on Notch signaling may underlie its putative involvement not only in cardiac diseases but also in tumor types induced by Notch deregulation, among whom are hematopoietic tumors [31, 32].

KCTD10, complexing with Cul3, also mediates the degradation of CEP97 protein, which is involved in blocking unscheduled ciliogenesis in proliferating cells [33, 34]. This observation suggests that KCTD10 defects may lead to a plethora of developmental defects, ciliary diseases and tumors caused by deregulation of pathways in which the primary cilium plays a role [35], including the potentially oncogenic Hedgehog (Hh) signaling pathway [36].

On the other hand, it has to be noted that KCTD10 expression has been suggested as a favorable prognostic marker in patients affected by gastrointestinal stromal tumor [37], and in hepatocellular carcinoma (HCC) cells. Indeed, Cul3/KCTD10 binds to and leads to degradation of Eukaryotic Translation Initiation Factor 3 subunit D (EIF3D), thus inhibiting cell growth [38].

Taken together, the available information seems to indicate a context-dependent function of KCTD10 in tumorigenesis, probably associated with the wide range of potential targets, and their relative concentration and activity in different cellular and tissue contexts.

***TNFAIP1*** is differentially regulated in a tissue-specific manner during mouse embryos development: particularly, it presents a high expression in brain, liver and heart tissues [39]. The gene is highly expressed in normal cell lines and is downregulated in cancer cell lines [40]. Indeed, in HCC cell lines, TNFAIP1 suppresses cell proliferation, metastasis, angiogenesis [41]. Several microRNAs have been described to negatively regulate TNFAIP1 in a large number of tumors, confirming its pivotal role of tumor suppressor: miR-372 and miR-373 in gastric carcinoma [42–45], miR-224 and miR-424 in non-small cell lung cancer (NSCLC) [46, 47], miR-181a in pancreatic cancer [48].

TNFAIP1 is frequently downregulated in uterine cancer tissues, and upregulation of TNFAIP1 expression inhibits tumorigenicity and cell growth in human cervical carcinoma (HeLa) and endometrial carcinoma cell line [49].

Analysis of COSMIC db suggest that lower levels of TNFAIP1 are also present in 14% of adrenal gland, data confirmed by analysis of GENT2 db (FC = 0.85; *p* value < 0.05).

In HeLa cells, TNFAIP1 overexpression and interaction with RhoB induces cell apoptosis via SAPK/JNK-mediated signal pathway [50]. Therefore, the capability to bind RhoB appears to be shared between KCTD10 and TNFAIP1, although the effects of this interaction may be different, suggesting the possibility of either a competition between the two proteins, or a partial redundancy of their functions.

Interestingly, KCTD10 and TNFAIP1 are also able to heterodimerize and TNFAIP1 has been shown to promote the proteasomal degradation of KCTD10 and inhibition of the transcriptional activities of NF-κB and AP-1 [51].

On the other hand, knockdown of TNFAIP1 in osteosarcoma (OS) cells repressed cell proliferation and invasion, and induced cell apoptosis, together with the downregulation of NF-κB signaling [43]. In the OS context, TNFAIP1 expression was significantly increased compared with adjacent non-cancerous tissues and positively correlated with metastasis [43]; targeting the 3’-UTR of TNFAIP1 mRNA by miR-15 has been suggested as a novel therapeutic strategy in this context [52].

Moreover, it was found that TNFAIP1 overexpression was related to an unfavorable prognosis in breast cancer [53] and TNFAIP1 has been suggested as a valid therapeutic target for the treatment of cancer with paclitaxel resistance, since it confers acquired resistance to this potent antitumor agent in various human malignancies [42].

***KCTD13*** is the least studied member of the clade C and its role remains largely elusive. Although it shares common features with its paralogs, it has been mainly described in neuropsychiatric and autism spectrum disorders, rather than pathological and cancerous status [54, 55].

KCTD13 has been recently demonstrated to target adenylosuccinate synthetase (ADSS) for ubiquitination and degradation [56]. ADSS is an enzyme that catalyzes the first step in adenosine monophosphate (AMP) synthesis. Of note, ADSS deletion has been previously highlighted in lung adenocarcinoma [57]. Based on these observations it can be hypothesized that ADSS reduction following overexpression of KCTD13 may favor cancerogenesis. Indeed, data extrapolated by us from COSMIC database suggest a positive role for KCTD13 in breast tumor, since its overexpression is found in 14% of samples analyzed and GENT2 db analysis confirmed this data (FC = 1.85; *p* value < 0.001).

## Group B: suppressors of Hh-dependent tumorigenesis

Group B is composed of KCTD11, KCTD21 and KCTD6 proteins. These proteins seem to have a more straightforward role in tumor suppression, since they share structural and functional features in control of the developmental and tumorigenic processes driven by Hh signalling. For this reason, the group B of KCTD proteins has recently been renamed as KCASH (KCTD Containing-Cul3 Adaptors, Suppressors of Hedgehog) family of proteins: in particular, KCTD11 (also known as REN) has become KCASH1, KCTD21 is now KCASH2 and KCTD6 as KCASH3 [12]. Indeed, these proteins may act in concert to negatively regulate the Hh signalling: binding to Cul3, they promote the ubiquitination and degradation of the Histone Deacetylase 1 (HDAC1), leading to hyperacetylation of Gli1 (the main transcription factor of the Hh signalling), and thus blocking its transcriptional activity [12, 13].

***KCASH1***^***KCTD11***^, the first member of KCASH family, maps on the chromosome 17p13.2 and its expression promotes growth arrest, differentiation, apoptosis and antagonizes Hh signalling in cerebellar granule cell progenitors [58, 59].

Interestingly, among the genetic alterations observed in Hh-dependent medulloblastoma (MB, cerebellar tumor), the most frequent one is represented by allelic deletion on chromosome 17p, occurring in up to 50% of tumors and frequently restricted to 17p13.2–13.3.19 region, where *KCASH1*^*KCTD11*^ maps [60]. Furthermore, KCASH1^KCTD11^ has been shown to be frequently down-regulated also by epigenetic modifications both in MB and prostate carcinoma [59, 61, 62].

A significant reduction of KCASH1^KCTD11^ expression has also been observed in other different types of human cancers: larynx, esophagus, stomach, colon-rectum, urinary bladder, lung, breast, gallbladder and endometrium [61]. Finally, in HCC tumors KCASH1^KCTD11^ inhibits cell proliferation and migration in vitro and tumor growth and metastasis in vivo [63]: indeed, it induces the G1/S cell cycle arrest through the p21 activation and repression of cycle-related proteins expression and inhibits tumor metastasis, by repressing EMT (epithelium-mesenchymal transition).

Finally, analysis of the COSMIC db shows a previously undescribed reduction of KCASH1^KCTD11^ expression in 59.8% of ovary cancer and GENT2 db confirmed this observation (FC = 0.82; *p* value < 0.05). Since the Hh pathway plays a role in the development and differentiation of the internal organs and its activation has been found in ovarian tumorigenesis [64], it would not be surprising if KCASH1^KCTD11^ had a role, direct or not, in ovarian maturation and malignancy. Hitherto, evidence of KCASH1^KCTD11^’s involvement in ovarian differentiation comes from studies in rat where the authors have observed that KCASH1 induction may be important for theca and granulosa cell differentiation into luteal cells [65].

***KCASH2***^***KCTD21***^ and ***KCASH3***^***KCTD6***^ analogously to KCASH1^KCTD11^, present high expression in cerebellum. The expression levels of *KCASH2*^*KCTD21*^ and *KCASH3*^*KCTD6*^ genes are significantly downregulated in the group of MB which are characterized by increased Hh signalling (Group SHH; [12, 15, 66]).

In addition to still unidentified epigenetic silencing events, *KCASH2*^*KCTD21*^ allelic deletion was also observed to contribute to the reduced expression of this gene [12]. Of note, chromosome 11q, where *KCASH2*^*KCTD21*^ gene is localized, can be lost in several sporadic tumors, including MB [67, 68], neuroblastoma [69], leukemia [70] and prostate cancer [71].

The role of KCASH3^KCTD6^ in Hh tumorigenesis seems to be less critical. Unlike its paralogs, KCASH3^KCTD6^ needs to heterodimerize with KCASH1 to downregulate the Hh signalling, since it is not able to directly bind HDAC1 [12].

KCASH3^KCTD6^ appears also to play other functions, being able to bind and lead to mono-ubiquitination the centrosome-associated deubiquitylase USP21. It has been demonstrated that USP21 recruits and stabilizes phosphorylated-Gli1 at the centrosome, where it could pilot the entry into or the exit of Gli1 from the primary cilium [72]. However, the mechanism underlying the role of KCASH3^KCTD6^ -USP21 complex on the Hh signalling regulation needs to be further explored.

Although it is likely that the KCASH family members present not only redundant functions, but also specific functions not yet identified, KCASH1^KCTD11^ and KCASH2^KCTD21^ appear as good candidates for novel biomarkers, and the design of methods to reactivate or increase their expression in tumor cells may be useful for treating different type of cancers.

To this end, the recent discovery of KCTD15 as a partner of KCASH2^KCTD21^, which stabilizes the protein and increases its activity, seems to be a promising result ([15] and see below). Furthermore a recent work has demonstrated the potential transcriptional modulation of KCASH2 by Sp1 and p53 [73].

**Fig. 1:**
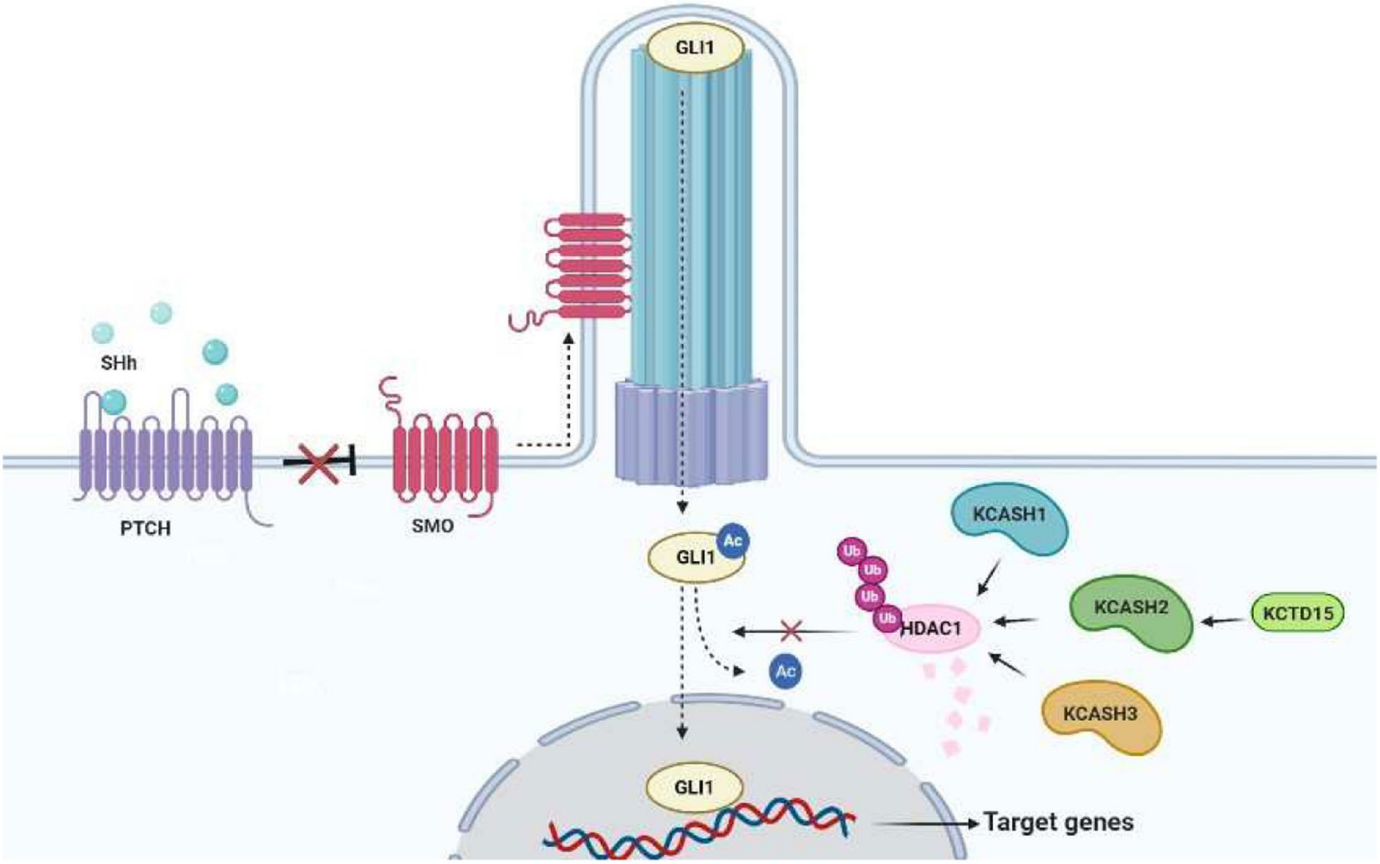
Group B KCTDs (KCASH family) suppress Hh/Gli1-dependent tumorigenesis. An example of cooperation between KCTD members. The Hh pathway is activated in the presence of SHh ligand, which binding to PTCH abolishes PTCH-mediated SMO inhibition allowing SMO to translocate into the primary cilium. Subsequently, ciliary trafficking of Hh pathway components leads to activation and transport of the transcription factor Gli1 towards the nucleus, where it transcribes target genes involved in proliferation and tumorigenesis. Along the way, Gli1 is kept transcriptionally active by HDAC1 deacetylation. The three members of the KCASH family act as negative regulators, promoting the ubiquitination and proteasomal degradation of HDAC1, thereby inhibiting transcriptional activity of Gli1. In this context, another KCTD protein, KCTD15, positively regulates KCASH2^KCTD21^ protein levels, enhancing its inhibitory activity. SHh: Sonic Hedgehog. PTCH: Patched

The role of the group B family members in the control of the Hh pathway is depicted in Fig. 1.

## Group A

Group A is composed by KCTD15 and KCTD1.

The ***KCTD15*** gene maps to chromosome 19q13.11 and is expressed in several tissues, including spleen, kidney, adult brain, and cerebellum [74, 75].

KCTD15 protein has been attributed to non-protein degradation functions. In fact, it was observed that KCTD15 is not able to interact with Cul3 and does not appear to be able to directly ubiquitinate and degrade its interactors [14].

KCTD15 has been previously suggested, by genome wide association studies (GWAS), to play a role in obesity and eating disorders [76, 77], although other groups did not confirm this association [78, 79]. On the other side, interaction between GRP78, a fundamental player in adipogenesis, and KCTD15 has been described [80].

Recently, a role for KCTD15 in cancer has been unveiled, based on its interaction with and stabilization of KCASH2^KCTD21^ protein [15]. In this way, KCTD15 enhances KCASH2^KCTD21^ inhibitory effect in MB cells, reducing tumor cell proliferation. Coherently, KCTD15 expression is reduced in a percentage of human sporadic MB which present Hh hyperactivation [15].

On the other hand, Smaldone and colleagues have shown that KCTD15 is upregulated in B-cell type acute lymphoblastic leukemia (B-ALL) patients [80] and acute myeloid leukemia (AML) patients [80, 81].

**KCTD1** is highly expressed in mammary glands, kidney, brain, and ovary [82].

KCTD1 was first identified as a transcriptional repressor, which repressed AP-2alpha-mediated transactivation through the BTB domain [82, 83]) and had the capability of binding to AP2α and removing it from the nucleus [84]. Subsequently KCTD1 was identified as the cause of Scalp-ear-nipple (SEN) syndrome, a rare autosomal-dominant disorder [17].

Although KCTD1 shares several features and biological functions with KCTD15, including the inability to bind to Cul3 [9], its role in cancer has still not been intensively investigated. However, unlike KCTD15, it has been shown that KCTD1 is involved in proteasomal degradation processes, by complexing with other E3-ubiquitine ligases, through which it participates in negative regulation of pathways which are aberrantly activated in many human cancers [85]. Indeed, KCTD1 has been proposed as an inhibitor of the WNT/β-catenin developmental pathway [85]: KCTD1 binds, through BTB domain, β-catenin, triggering its cytoplasmic accumulation. Here, KCTD1 contributes to an overall decrease of β-catenin protein levels, via β-TrCP-mediated proteasome pathway, dependent on the phosphorylation of the Ser45 and Ser33/Thr41 sites mediated by CK1 and GSK-3β kinases. Interestingly, ectopic expression of KCTD1 in cervix tumor HeLa cells inhibits the expression of β-Catenin downstream target genes and while APC counteracts the KCTD1-mediated downregulation of β-catenin, p53 enhances KCTD1 inhibitory role [85].

Therefore, it is likely that KCTD1 may play a oncosuppressive role by promoting the degradation of β-catenin, a core player in canonical WNT/β-catenin signaling pathway [85].

Intriguingly, evidence from COSMIC and GENT2 db, suggest a potential pro-tumorigenic role of KCTD1, which is overexpressed in subsets of ovary tumors (13.9% of tumor analyzed, FC = 1.47, *p* value < 0.001), pancreatic cancer (11.17% of samples; FC = 1.32, *p* value < 0.001), endometrium tumor (9.8%; FC = 1.43, *p* value < 0.001), lung tumor (7.46%; FC = 1.21, *p* value < 0.001).

The high homology of sequence between KCTD1 and KCTD15, both at level of the BTB domain and of the full sequence, would have suggested similar mechanisms of action. Surprisingly while KCTD1 enhances β-catenin degradation by the β-TrCP-mediated proteasome pathway [85], KCTD15 is involved in KCASH2^KCTD21^ protein stabilization. Of course, even though KCTD15 cannot bind Cul3, [14, 15] we cannot exclude the possibility that also KCTD15 may bind other E3 ligases in different contexts. On the other hand, it cannot be ruled out that also KCTD1 may stabilize specific proteins modulating tumorigenesis.

## Group D

Group D comprises SHKBP1 (also known as SETA binding protein 1, SB1) and KCTD3 proteins.

**SHKBP1** biological function has not been completely characterized, although several hints suggest a significant role in tumorigenesis.

SHKBP1 presents a 55% aa identity to the renal tumor antigen NY-REN-45 and was initially identified as a protein that binds to SH3 domains of the SETA adapter protein, whose hyperexpression has been associated with adult brain tumors [84]. In this context, it is interesting that our analysis of COSMIC db shows that 5.4% of nervous system tumors presents SHKBP1 mutations.

SHKBP1 expression levels are also increased in osteosarcoma (OS) samples compared to their normal counterparts [86]. In this context, SHKBP1 may promote EGFR signaling pathway by interrupting c-Cbl-CIN85 complex and inhibiting EGFR degradation [87].

Furthermore, serum protein profiling identified SHKBP1 as a candidate in earlier detection of small intestine neuroendocrine tumor (WD-SI-NETs) tumor [88].

Moreover, whole genome sequencing identified a missense mutation of *SHKBP1* in acute myeloid leukemia (AML), which characterizes SHKBP1 as a putative proto-oncogene. In particular, the V891I mutation is suggested to interfere with binding to SETA, disturbing the degradation of the FLT3 tyrosine kinase [89]. Recently, through a comprehensive genomic study of cervical cancer, SHKBP1 has been also identified as a mutated gene in the squamous subtype [90].

Finally, analysis of COSMIC db suggests SHKBP1 overexpression in pancreatic tumor (13.4%; FC = 2.05, *p* value < 0.001 extrapolated by GENT2 db) and cervical cancer (10.8%; FC = 2.14, *p* value < 0.05 by GENT2 analysis). It is also interesting to note that 4.5% of large intestine cancer present SHKBP1 mutations.

While **KCTD3** has been associated with neurogenetic and neurodevelopmental disorders [1, 91], no correlation with cancer has been reported so far.

Nevertheless, KCTD3 has been demonstrated to interact and increase stability and cell surface expression of hyperpolarization-activated cyclic nucleotide-gated channel 3 (HCN3) [92]. Intriguingly, HCN3 has been found overexpressed in neuroblastoma tumors and has been indicated to protect cells from apoptosis driven by HIF-1a and p53 [93]. Furthermore, analysis of COSMIC db indicate that 4% of nervous system tumor samples present KCTD3 mutations, suggesting the need to verify KCTD3 role in this context.

Similarly to its paralog SHKBP1, analyses performed on COSMIC database point out KCTD3 overexpression in other tumor types, such as breast cancer (14.2%; FC = 1.3, *p* value < 0.001 in the GENT2 db) and stomach (13%; FC = 1.83, *p* value < 0.001 by GENT2 analysis).

## Group F: not only neurodegenerative and neuropsychiatric disorders

KCTD12, KCTD8 and KCTD16 proteins belong to the F clade of the KCTD family and are known as auxiliary subunits of the GABA_B1/2_ receptor (G-protein coupled receptors for GABA, the main inhibitory neurotransmitter in the central nervous system [94]).

Given the role they play in regulating GABA receptor signalling, mutations in *KCTD12*, *KCTD8* and *KCTD16* genes have been implicated in neurodegenerative and neuropsychiatric disorders [1].

Among these proteins, **KCTD12** has also been linked to tumorigenesis, in some contexts as a potential oncosuppressor, in other contexts as oncogene. *KCTD12* (also known as Pfetin) was identified as a gene predominantly expressed in fetal tissues [95] and suggested as a biomarker for the diagnosis and prognosis of gastrointestinal stromal tumors (GIST). In particular, a decreased pfetin expression was observed in a subset of GISTs with poor clinical outcomes and pfetin expression significantly affected the disease-free and overall survival of the patients [96, 97].

In colorectal cancer (CRC) cells down-regulation of KCTD12 leads to an increase of staminality markers [98]. The KCTD12 inhibitory role on stemness has also been confirmed in vivo through xenotransplantation experiments [98].

KCTD12 displays a reduced expression in breast cancer tissues and cells, correlated with patients’ overall poorer survival. Indeed, downregulation of KCTD12 significantly promotes cancer cell proliferation and G1/S transition through the AKT/FOXO1 signaling [99]. Consistently, ectopic expression of KCTD12 in human uveal melanoma cells causes a retention in the G2/M phase, an inhibition of proliferation and an increase of apoptosis, which has been confirmed in vivo through xenograft experiments [100]. Moreover, KCTD12 acts as a tumor suppressor also in esophageal squamous cell carcinoma (ESCC), downregulating WNT and NOTCH signaling [101].

In other contexts, KCTD12 may promote cell proliferation: increased expression of KCTD12 has been observed in cervical, colon and lung cancers and its high levels have been correlated with poor prognosis [102, 103]. In fact, KCTD12 interacts with CDK1 and CDC25B, forming a complex that supports CDK1 phosphorylation and cell cycle progression [103], while pharmacological inhibition of KCTD12-CDK1 interaction suppresses growth of colon cancer cells in vitro and in vivo [102].

**KCTD16** is a transcriptional target of ASCL1, one of the master transcription factors of small cell lung carcinoma, (SCLC) [104]. Comparative RNA-seq studies and immunohistochemical staining revealed a strong KCTD16 expression in H69 cells (ASCL1-positive, classical type SCLC) and in ASCL1-transfected A549 adenocarcinoma cell lines, while in H69AR (ASCL1-negative, variant type SCLC) and A549 (control) cell lines the level of KCTD16 protein does not result upregulated [104]. The definition of the biological significance of KCTD16 upregulation in neoplastic development requires further investigation.

*KCTD16* expression has been suggested as a negative prognostic marker in thyroid cancers [105]. On the other side, analysis of the COSMIC db indicates that *KCTD16* presents mutations in hepatic tumors (13.6% of samples), pancreas (11.7%) and prostate (8.9%), suggesting the need to evaluate if these mutations are inactivating or hyperactivating the protein.

Almost unexplored is the potential role of **KCTD8** in cancer. Hypermethylation and transcriptional repression of *KCTD8* was observed in breast cancer samples [106]. On the other hand, COSMIC db analysis suggests high KCTD8 expression in thyroid cancer (8% of samples analyzed), pancreas (7.3%), esophagus (7.2%) and cervix (5.5%).

## Insides into the role of E, G, H groups and other KCTD proteins in cancer

KCTD2, -5, -9, -17 proteins belong to group E. For all these proteins, there is still little information and their role in physiological and pathological contexts and even less is known about their involvement in the onset or progression of cancer.

The most studied protein of this clade is undoubtedly **KCTD5** the only member of the KCTD family whose three-dimensional structure has been resolved. Indeed, KCTD5 presents a pentameric association of both the BTB and the C-terminal domains and a central spanning cavity [107]. KCTD5 and KCASH3^KCTD6^ share a high sequence identity (> 40%) at BTB domain [108]. Like KCASH3^KCTD6^, KCTD5 is able to bind Cul3 [109].

KCTD5 has been suggested to act as a negative regulator of cell migration by modulating cell spreading and focal adhesion dynamics. Indeed, KCTD5 is a negative regulator for the migration of melanoma and breast cancer cells, by affecting Rac1 activity and Ca^2+^ signaling [110]. Given these data, KCTD5 could play a role in inhibition of processes associated with cancer metastasis.

On the other side, KCTD5 positively regulates the Transient Receptor Potential Melastatin 4 (TRPM4) [111]. Since TRPM4 activity has been proposed to contribute to the pathophysiology of different cancers, and TRPM4 and KCTD5 expression are increased in breast cancer (in particular in the most aggressive subtype, the triple negative one), study of the TRPM4-KCTD5 protein interaction could be useful to develop drugs that modulate their activity with therapeutics purposes [111].

Confirming these data, COSMIC and GENT2 db indicate KCTD5 overexpression in 12.5% of breast cancer samples (FC = 1.33, p-value < 0.001). It would be interesting to further evaluate if KCTD5 is restricted to non metastatic breast tumors, as it could be suggested by its role on TRPM4.

Further involvement of KCTD5 in other tumor types has not yet been described, but Brockmann and colleagues demonstrated that KCTD5 may switch off the Akt pathway, which regulates cell survival, cell cycle progression and cellular growth [112]. Furthermore, since the AKT pathway interplays with Hh signaling through the non-canonical pathway, it may be possible that KCTD5 alterations affect the Hh signaling-dependent tumorigenesis [113, 114].

Evidence of **KCTD2** involvement in cancer derive from studies conducted on glioblastoma tumor cells and CRC cancer patients. Indeed, KCTD2 promotes ubiquitination and degradation of the oncogene c-Myc, and reduced KCTD2 mRNA levels are present in glioma cells, promoting tumor growth in vivo [8]. On the other hand, microarray analysis suggests that KCTD2 may be upregulated in colorectal cancer [115].

**KCTD17** protein controls ciliogenesis through polyubiquitylation and subsequent degradation of trichoplein, a keratin-binding protein that controls the recruitment of microtubules to centrioles, therefore participating in the initial step of axonemal extension during ciliogenesis [116].

Knockdown of KCTD17 stabilizes trichoplein which in turn binds Aurora A and elevates its kinase activity. In turn Aurora A destabilizes axonemal microtubules, resulting in the deciliation at the cell cycle reentry or the inhibition of unscheduled ciliary assembly, affecting ciliary dynamics in development and tissue homeostasis. [117]. KCTD17 is therefore likely to play a role in tumorigenesis, given the role of cilia in transmission of environmental and intracellular signals, both repressive and promoter of proliferation and control of cell cycle [118].

Indeed, recently has been demonstrated that depletion of Trichoplein expression results in chromosome mis-segregation, DNA damage and chromosomal instability in cancer cells [119].

**KCTD9** is the only human protein containing five pentapeptide repeats (presumably a DNA mimicking structure) [6], which is described to play a role in NK cell lineage commitment and maturation, mediating by direct or indirect interaction-dependent degradation of transcription factors or chromatin regulators a marked increase in CD69 expression, cytotoxicity, IFN‐γ secretion and a significant decrease in NKG2A receptor expression (120).

No information has been published yet on KCTD9 and cancer, although we would expect a role for KCTD9 in tumorigenesis, given that NK cells are a key immune constituent in the protective antitumor immune response [121]. Of note, COSMIC and GENT2 db analysis indicate KCTD9 overexpression in 14.7% of gastric tumor samples and FC = 1.25 (*p* value < 0.05) respectively.

**KCTD20** and **BTBD10** belong to group G. The amino acid sequences of these two proteins present a high homology, especially in correspondence of the C-terminal region, which suggests that BTBD10 and KCTD20 share functional characteristics and can play similar biological role. Indeed, both proteins are involved in the activation of Akt signaling [122, 123] and it is therefore possible that aberrant expression of these genes can be implicated in tumorigenic processes.

A recent study correlates high KCTD20 expression with advanced non-small cell lung cancer (NSCLC), characterized by positive regional lymph node metastasis and poor prognosis [113]. Indeed, it has been observed that KCTD20, through activation of the Akt signaling, increases cell proliferation and facilitate the invasion of NSCLC cells by inhibiting E-cadherin expression (113).

Similarly, BTBD10, also known as glucose metabolism-related protein 1 (GMRP1), inhibits apoptosis of neuronal and islet β-cells via Akt pathway. On the other hand, another group indicates that BTBD10 is downregulated in glioma tumor, suggesting that it might play a negative role in the proliferation and progression of glioma cells [120, 123].

KCTD7 and KCTD14 have been clustered in group H, according to their sequence homology, but have not been characterized yet.

**KCTD7** alternative splicing has been suggested to have prognostic value in glioblastoma [124] and lung adenocarcinoma [125]. Analysis of GENT2 and COSMIC Db indicate that KCTD7 is upregulated in tumors affecting the following tissues: adrenal gland (13.9%; FC = 1.61, p-value < 0.001 by GENT2 db), skin (8.46%, coherent with GENT2 FC = 2.28, *p* value < 0.001), breast (6.16%; FC = 1.16, *p* value < 0.001 by GENT2 analysis). Moreover, 5.5% of the pancreatic tumors analyzed (COSMIC) present a KCTD7 mutation.

**KCTD14** has not so far been investigated, and involvement in the onset and progression of cancer has not been documented, DB analysis suggests a potential protumor role in ovary cancers: COSMIC reports a CNV gain in 4.5% of ovary cancers and GENT2 a FC = 1.5 (*p* value < 0.001) in expression.

The KCTD proteins that are not classified in any group, since they do not share homologies with other KCTD members, are in general not characterized.

Among them, **KCNRG** is the only one whose role in cancer is known, albeit partially.

The human *KCNRG* is located on 13q14.3 and was described as a K+ channel regulator [126].

KCNRG overexpression in leukemia cell lines exerts growth suppression, promotes apoptosis, leads to changes in size and shape, and causes the decrease of cell migration capacity [126]. Interestingly, deletion of 13q14 is the most frequent chromosomal anomaly in chronic lymphocytic leukemia (CLL). Rearrangements and/or deletions in the 13q14.3 region were found also in other types of hematopoietic neoplasms, including 38% of mantle cell lymphomas and approximately 54% of multiple myelomas (MM). In most of these non-CLL cases, 13q14 deletions were associated with a poor prognosis [127]. These data suggest that KCNRG can act as tumor suppressor and may play a relevant role in the pathogenesis of CLL and MM tumors.

13q14.3 deletions are also common in prostate cancer and gastrointestinal stromal tumors. Sequencing analysis of 77 Hepatocellular carcinomas (HCCs) showed that KCNRG can be mutated in HCC tumors. This missense mutation is caused by Arg to His substitution at codon 92 in exon 1 encoding the KCNRG BTB domain. Similarly to leukemia cells, KCNRG overexpression suppresses the growth activity of Hep3B hepatoma cells while its inactivation, through mutations or allelic losses, contributes to the development or progression of HCC [128].

**KCTD19**, so far observed mutated only in 44% of patients affected by Cerebral Visual Impairment [129], is found upregulated in hematopoietic and lymphoid district tumors (12.7%; FC = 1.14, p-value < 0.001). Similarly, KCTD19 is mutated in cancer tissues from nervous system (4.7%), large intestine (3.2%) and skin (4.2%).

**KCTD4** has not been characterized. Db analysis indicate is overexpressed in 5% of sample of analyzed tumor associated to lung cancer (GENT2 db: FC = 1.32, p-value < 0.001).

Finally, **KCTD18** has been only potentially associated to Restless Legs Syndrome [130] and no information are available that link KCTD18 to tumor development. Since the protein structure has not been defined, no inference can be done on its function. Also, analysis of the db did not provide univocal indications or correlations with tumor types.

## Conclusions

The family of KCTD proteins is formed by 25 members, with most of them still poorly characterized.

Over time, some of the KCTD have been associated with neurodevelopmental and neuropsychiatric disorders, obesity, or modulation of signal transduction pathways, while information of their role in cancer is scattered within the literature.

Furthermore, analysis of the KCTD family is complex, since homology between these proteins is often limited to the conserved BTB domain, and these proteins, even when members of the same groups, may be involved in rather different pathological processes. This can in part due to the roles played by some of these proteins in ubiquitination and degradation of a wide plethora of different targets. Furthermore, some of the characterized (and most of the uncharacterized) members of the family are likely involved in other basic processes that are yet to be identified, including fundamental biological processes that can play a role also in tumorigenesis.

Cancer is considered the leading cause of death worldwide. Genetic and histological heterogeneity of cancers, the ability of cancer cells to evade the immune response and to remodel pleiotropic systems contribute to the difficulty of understanding how different cancer develops, grows, metastasizes and identifying valid therapeutic strategies that do not lead to resistance and relapse. In the era of precision medicine, the knowledge of all perturbations of molecular pathways and identification of all the genes playing a role in tumorigenesis has become more necessary than ever, both for therapeutic purposes and for diagnosis and prognosis.

**Fig. 2:**
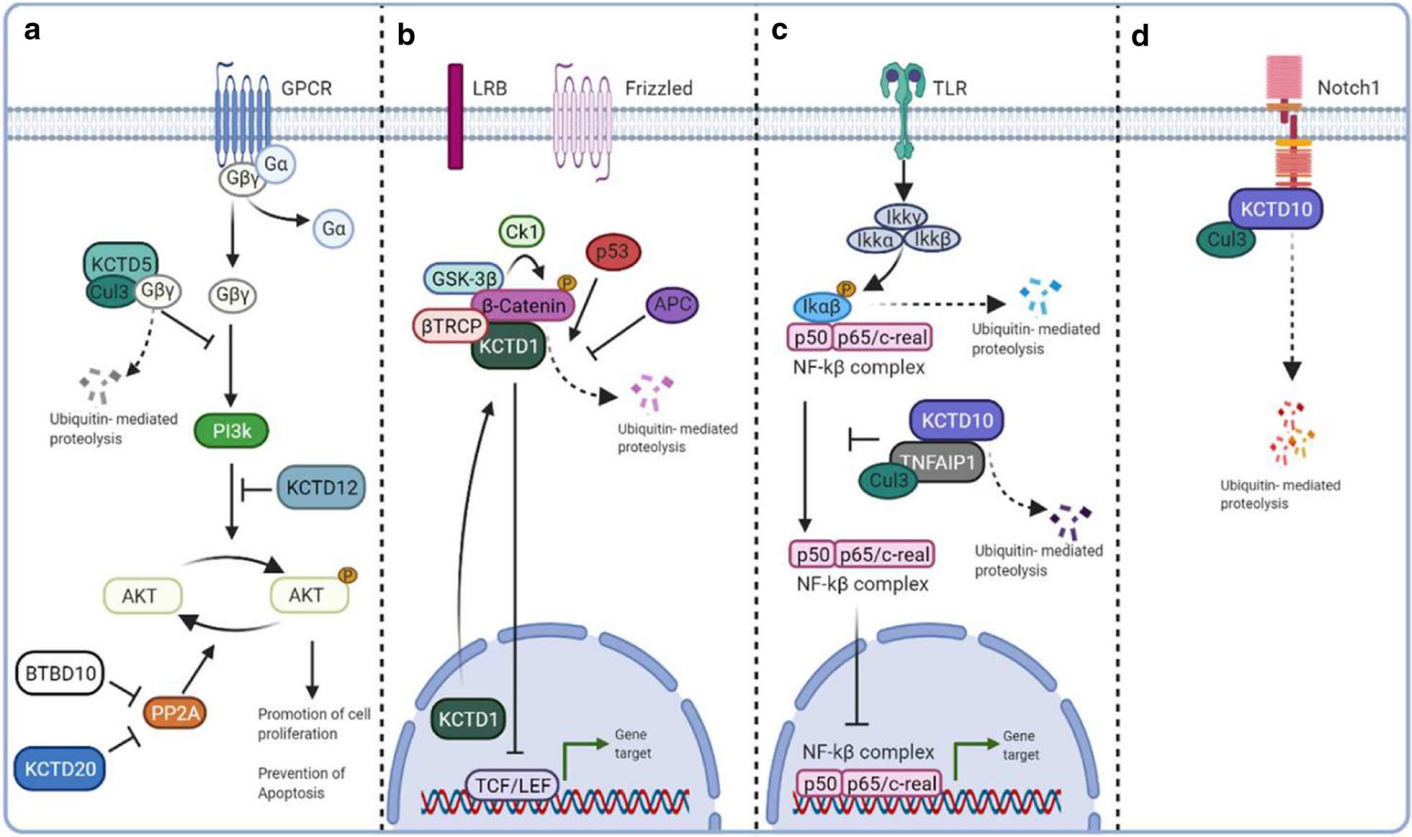
Schematic representation of the role of KCTDs in modulation of potentially tumorigenetic pathways. **a**: PI3K/AKT pathway. The activation of the GPCR receptor leads to the dissociation of the heterotrimer G into Gα and Gβγ. Subsequently, Gβγ activates PI3K that phosphorylates AKT. KCTD5 and KCTD12 negatively regulate the AKT pathway by triggering proteolysis of Gβγ heterodimer and inhibiting the phosphorylation of AKT, respectively. KCTD20 and BTBD10 suppress the PP2A-mediated dephosphorylation of AKT, acting as positive regulators of the pathway. **b**: WNT pathway. In the absence of Wnt ligand, β-catenin is degraded through the “destruction complex” (composed of CK1, axin, GSK-3β and APC), which phosphorylates β-catenin for ubiquitin-mediated proteasomal degradation. KCTD1 negatively regulates Wnt signalling by Enhancing β-catenin degradation. **c**: NF-κB pathway. Binding of inflammatory cytokines to TLR leads to the activation of the IKK complex, which phosphorylates IκBα. IκBα phosphorylation induces its ubiquitination and degradation by the proteasome, leading to nuclear translocation of the NF-κB transcriptional complex. TNFAIP1 interacts with KCTD10 inducing its degradation and inhibits the transcriptional activity of NF-κB. **d**: Notch pathway. KCT10 interacts with Cullin3 and the intracellular domain of Notch1, mediating Notch1 ubiquitination and proteolytic degradation

Few of the KCTD groups have been identified as key players in the modulation of specific tumorigenic pathway (e.g., the KCASH family; see Fig. 1), but the collection of all the evidence available up to now allow us to hypothesize that the number of KCTD family member involved in tumorigenesis (either as positive or negative modulator) may be far bigger than so far demonstrated. Figure 2 summarizes the available data on the role of KCTDs in modulation of other potentially tumorigenetic pathways**.** Homology between family members, capability to participate in ubiquitination and degradation of different targets and to take part to the signal transduction of developmental and tumorigenic pathways, ability to heterodimerize between members, bioinformatic analysis of available databases in the search for positive or negative correlations with cancers, are all factors that need to be considered in the search for new functions in this family. In this review we referred to COSMIC and GENT2 db, two user-friendly search platform for gene expression patterns across different normal and tumor tissues. The gene expression profiles generated by a large number of samples (68,000 for GENT2 [20]; and genome-wide analysis of over 37,000 genomes for COSMIC2) may provide an extensive coverage of the cancer genomic landscape.

**Fig. 3:**
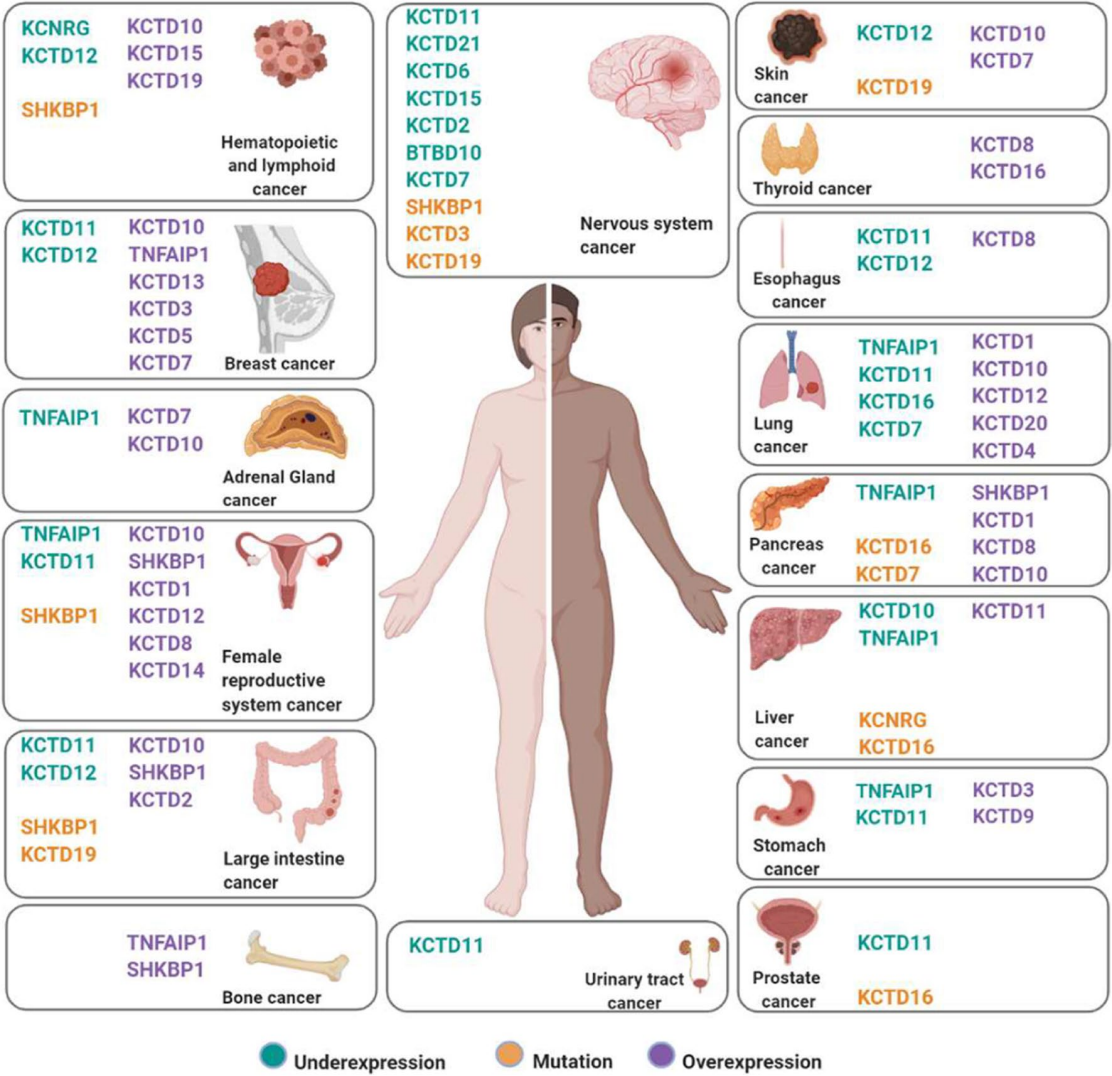
The KCTD family in human cancers. Synthesis of the current knowledge on the role of KCTD proteins in different cancer types. KCTDs whose expression is downregulated in cancer are in green; mutated KCTDs are in orange; KCTDs which are overexpressed in violet. All information is the result of data extrapolated by literature and analysis of COSMIC-GENT2 db, details are in the text

In order to provide new hints to the scientific community we have summarized in this review most of the published material integrated with data extrapolated from COSMIC and GENT2 databases (summarized in Fig. 3), whose knowledge could be a good starting point for further necessary research.

## Data Availability

The datasets generated and/or analyzed during the current study are available in the following repository: COSMIC (Catalogue Of Somatic Mutations In Cancer; https://cancer.sanger.ac.uk/cosmic and GENT2 (Gene Expression patterns across Normal and Tumor tissues; http://gent2.appex.kr/gent2/.

## Abbreviations

AML: Acute Myeloid Leukemia
BTB/POZ: Bric-a-brac, Tramtrak, Broad complex/ Poxvirus zinc finger
CLL: Chronic lymphocytic Leukemia
CNV: Copy Number Variant
COSMIC: Catalogue Of Somatic Mutations In Cancer
CRC: Colon-Rectal Cancer
CRL: Cullin Ring Ligase
Cul3: Cullin 3
Db: Database
EIF3D: Eukaryotic Translation Initiation Factor 3 subunit 3D
EMT: Epithelial–Mesenchymal Transition
ESCC: Esophageal Squamous Cell Carcinoma
FC: Fold Change
GENT2: Gene Expression patterns across Normal and Tumor tissues
GIST: Gastro Intestinal Stromal Tumors
GMRP1: Glucose Metabolism-Related Protein 1
GWAS: Genome Wide Associations Studies
HCC: HepatoCellular Carcinoma
Hh: Hedgehog
KCASH: KCTD containing Cul3 Adaptors Suppressor of Hedgehog
KCTD: Potassium (K+) Channel Tetramerization Domain
MB: Medulloblastoma
MM: Multiple Myelomas
NSCLC: No Small Cell Lung Cancer
OS: Osteosarcoma
PCNA: Proliferating Cell Nuclear Antigen
PDIP1: Polymerase Delta-Interacting Protein 1
SCLC: Small Cell Lung Carcinoma
SNV: Single Nucleotide Variant
TNFAIP1: Tumor Necrosis Factor Induced Protein 1
TRPM4: Transient Receptor Potential Melastatin 4
Ub: Ubiquitination
